# Risk factors for different types of arrhythmias and their prognostic impact in patients with heart failure and concomitant pulmonary hypertension (PH-LHF): a large-scale retrospective cohort study

**DOI:** 10.3389/fcvm.2026.1773989

**Published:** 2026-04-15

**Authors:** Tian Dai, Bo Liu, Qian Zhao, Xiaoli Li, Haifeng Chen, Ping Yang

**Affiliations:** Department of Cardiology, The Sixth Hospital of Wuhan, Affiliated Hospital of Jianghan University, Wuhan, China

**Keywords:** arrhythmia, atrial fibrillation, heart failure, pulmonary hypertension, right ventricular dysfunction, ventricular tachycardia

## Abstract

**Background:**

Pulmonary hypertension with left heart failure (PH-LHF) is a prevalent phenotype with distinct hemodynamics. The arrhythmic landscape—encompassing atrial tachyarrhythmias (AT), ventricular tachyarrhythmias (VT), and bradyarrhythmias (BA)—remains poorly characterized. We aimed to identify phenotype-specific risk factors and prognostic impacts of these arrhythmias in PH-LHF.

**Methods:**

This retrospective cohort study included 1,530 PH-LHF patients. Patients were stratified into four mutually exclusive groups based on hierarchical rhythm documentation: No Arrhythmia (NA), AT, VT, and BA. Multivariate Logistic regression and Cox proportional hazards models (adjusting for NT-proBNP, ischemic etiology, and treating device therapy as time-dependent covariates) were utilized to determine independent predictors of each arrhythmia type and to assess their impact on all-cause mortality and heart failure rehospitalization over a median follow-up of 38 months.

**Results:**

The overall prevalence of clinically significant arrhythmias was 68.9%, comprising AT (42.2%), VT (18.0%), and BA (8.7%). Left atrial volume index (LAVI) >40 ml/m² (OR 2.41, 95% CI 1.85–3.15) was the strongest predictor of AT. Conversely, a marker of right ventricular dysfunction (TAPSE <16 mm) was a potent independent predictor of VT (OR 3.25, 95% CI 2.38–4.45), largely independent of LVEF. Prognostically, VT was associated with the highest risk of all-cause mortality (HR 2.56, 95% CI 1.95–3.36), whereas AT was the primary driver of rehospitalization (HR 1.92, 95% CI 1.62–2.28). The prevalence of VT increased disproportionately with PH severity. Overlap analysis revealed that 15% of patients in the VT group also had documented AT, and secondary time-dependent analyses confirmed the independent prognostic weight of VT.

**Conclusion:**

Arrhythmia burden in PH-LHF is substantial and biologically distinct. The “right heart phenotype” drives malignant ventricular arrhythmias and mortality, while the “left atrial phenotype” drives atrial arrhythmias and morbidity. These findings advocate for a precision medicine approach, suggesting that RV monitoring could serve as a valuable tool for risk enrichment in SCD risk stratification in this vulnerable population, pending prospective validation.

## Introduction

Heart failure (HF) and pulmonary hypertension (PH) constitute a dual epidemic in modern cardiovascular medicine. When they coexist, they form a specific clinical entity known as Pulmonary Hypertension associated with Left Heart Failure (PH-LHF), classified as Group 2 PH by the World Symposium on Pulmonary Hypertension (WSPH) (1, 2). With the aging global population, the prevalence of PH-LHF is rising, now complicating up to 70% of heart failure with reduced ejection fraction (HFrEF) and a comparable proportion of those with preserved ejection fraction (HFpEF) (3, 4). Unlike isolated left heart disease, PH-LHF involves a complex hemodynamic progression: initial passive backward transmission of elevated left ventricular filling pressures is frequently followed by a superimposed pre-capillary component—termed Combined Pre- and Post-capillary PH (Cpc-PH) (5). This transition is marked by pulmonary vascular remodeling, increased pulmonary vascular resistance (PVR), and eventual right ventricular (RV) uncoupling, leading to a significantly worse prognosis compared to isolated HF (3).

Arrhythmias are inextricably linked to the natural history of heart failure, serving as both a marker of disease severity and a driver of decompensation (6, 7). However, the current understanding of arrhythmogenesis in PH-LHF is fragmented. Existing guidelines and major clinical trials have largely treated “heart failure” as a homogeneous entity regarding rhythm management, or have focused narrowly on Atrial Fibrillation (AF) as the sole arrhythmia of interest (8, 9). This reductionist approach neglects the specific electromechanical substrate created by PH-LHF. Patients with PH-LHF are subjected to a unique “double hit” of remodeling: the left atrium is subjected to chronic stretch and fibrosis due to elevated filling pressures, predisposing to atrial tachyarrhythmias (AT) (6); simultaneously, the right ventricle faces chronic pressure overload, leading to hypertrophy, ischemia, and fibrosis, which may create a potent, yet under-recognized, substrate for ventricular tachyarrhythmias (VT) and conduction system disease (Bradyarrhythmias, BA) (8).

Recent translational and clinical data from 2023 to 2024 suggest that the presence of PH fundamentally alters the arrhythmic risk profile. For example, RV dysfunction has emerged as a potential independent predictor of sudden cardiac death (SCD), yet it remains absent from current primary prevention ICD guidelines, which rely almost exclusively on left ventricular ejection fraction (LVEF) (10, 11). We hypothesize that RV parameters may play a critical role in risk enrichment for these patients. Furthermore, the impact of PH on the conduction system is gaining attention, with hypotheses suggesting that mechanical strain on the interventricular septum may disrupt the bundle branches, leading to bradyarrhythmias that complicate beta-blocker therapy (1, 12). Despite these emerging concepts, there is a paucity of large-scale data stratifying the risk factors and prognostic implications of these distinct arrhythmia subtypes specifically within the PH-LHF population. Does the “failing right heart” specifically drive ventricular instability? Is the prognostic weight of new-onset AF different in a patient with severe PH compared to one without?.

To address these gaps, we designed this retrospective cohort study with a granular “phenotype-specific” approach. Unlike general HF studies, we isolated the PH-LHF cohort to: (1) determine the prevalence and distribution of specific arrhythmia subtypes (AT, VT, BA); (2) identify the unique clinical and echocardiographic risk factors driving each subtype, with a particular focus on RV parameters; and (3) quantify the independent contribution of each arrhythmia type to long-term mortality and rehospitalization. We hypothesized that distinct structural remodeling patterns (LA dominant vs. RV dominant) predispose to specific arrhythmias, and that these arrhythmias carry divergent prognostic implications that could inform more personalized therapeutic strategies.

## Methods

### Study design and ethical considerations

This single-center, retrospective cohort study was conducted at our hospital, a high-volume tertiary cardiovascular center. The study protocol complied with the ethical guidelines of the 1975 Declaration of Helsinki and was approved by the Institutional Review Board. The requirement for individual informed consent was waived due to the retrospective design and anonymization of patient data. The reporting of this study follows the STROBE (Strengthening the Reporting of Observational Studies in Epidemiology) guidelines (13).

### Study population and selection criteria

**Figure 1:**
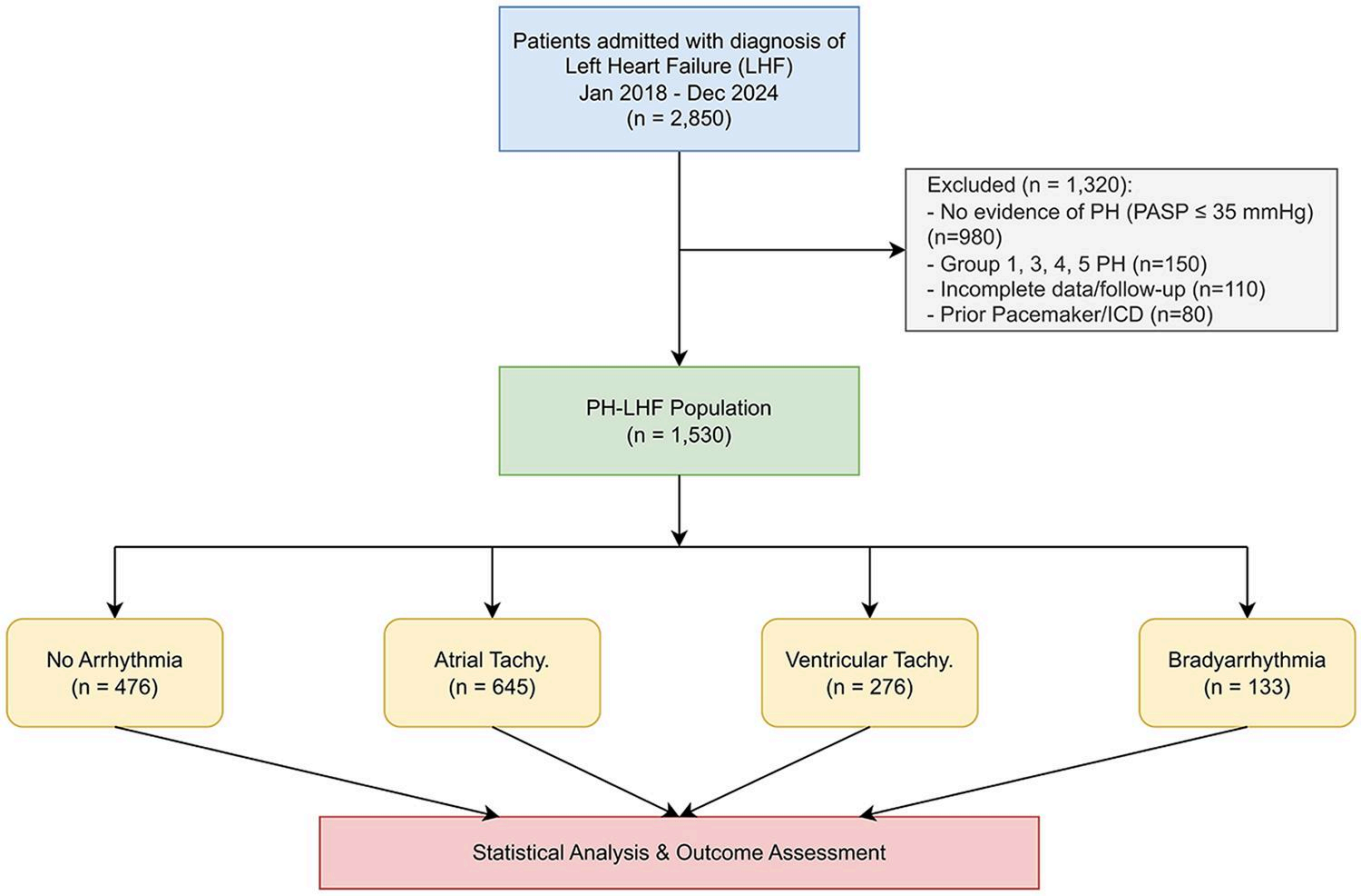
Study flowchart. Diagram illustrating the patient selection process, exclusion criteria, and final stratification into four arrhythmia subgroups (NA, AT, VT, BA).

We systematically screened the electronic medical record (EMR) system for all adult patients (aged ≥18 years) hospitalized with a primary diagnosis of Left Heart Failure (ICD-10 codes I50.1, I50.9) between January 1, 2018, and December 31, 2024. The selection process is detailed in Figure 1.

**Inclusion Criteria:** (1) Symptomatic heart failure [New York Heart Association (NYHA) functional class II-IV]; (2) Objective evidence of left heart disease (LVEF <50% or significant diastolic dysfunction); (3) Concomitant Pulmonary Hypertension, defined non-invasively by a Pulmonary Artery Systolic Pressure (PASP) >35 mmHg on echocardiography (14), or invasively by a Mean Pulmonary Artery Pressure (mPAP) >20 mmHg with Pulmonary Artery Wedge Pressure (PAWP) >15 mmHg (15, 16); (4) Availability of continuous electrocardiographic monitoring (telemetry for ≥24 h or 24 h-Holter) during the index admission.

**Exclusion Criteria:** (1) Pre-capillary PH (Group 1 PAH, Group 3 Lung Disease, Group 4 CTEPH) without significant left heart disease; (2) Acute coronary syndrome, cardiac surgery, or percutaneous coronary intervention within 90 days prior to admission; (3) Presence of an active cardiac implantable electronic device (pacemaker, ICD, CRT) prior to index admission, to accurately assess the incidence of intrinsic arrhythmias; (4) Severe non-cardiac comorbidities (e.g., active malignancy, end-stage liver disease) reducing life expectancy to <1 year; (5) Incomplete clinical or follow-up data.

### Arrhythmia classification and definitions

The primary exposure of interest was the type of arrhythmia identified during the index hospitalization or within 3 months post-discharge via ambulatory monitoring. Patients were categorized into four mutually exclusive groups based on a clinical hierarchy of severity (VT > AT > BA > None) to handle patients with multiple arrhythmias.

- **Ventricular Tachyarrhythmia (VT) Group:** Documentation of sustained VT (lasting >30 s), hemodynamically unstable VT of any duration, ventricular fibrillation (VF), or frequent non-sustained VT (NSVT). Clinically significant NSVT was strictly defined as episodes consisting of ≥3 consecutive ventricular beats at a rate >100 bpm that were symptomatic or associated with hemodynamic compromise (11, 17).
- **Atrial Tachyarrhythmia (AT) Group:** Documentation of Atrial Fibrillation (AF), Atrial Flutter (AFL), or focal Atrial Tachycardia lasting >30 s (18, 19).
- **Bradyarrhythmia (BA) Group:** Sinus node dysfunction (pause >3 s, symptomatic sinus bradycardia <50 bpm), second-degree type II or third-degree atrioventricular (AV) block, or bundle branch block requiring consideration for pacing. Bradycardia solely attributable to reversible causes (e.g., acute drug overdose) was excluded (20).
- **No Arrhythmia (NA) Group:** Patients without documentation of the above rhythm disturbances during the screening period.

### Data collection

#### Clinical variables

Baseline demographics, comorbidities [hypertension, diabetes, chronic kidney disease (CKD)], and medication usage were extracted. CKD was defined as an estimated glomerular filtration rate (eGFR) <60 mL/min/1.73m². Detailed characterization of heart failure phenotypes (HFrEF, HFmrEF, HFpEF), etiology (ischemic vs. non-ischemic), and pharmacological therapies (including beta-blockers, antiarrhythmics, and SGLT2 inhibitors) have been incorporated to provide broader clinical context.

#### Echocardiographic assessment

All patients underwent comprehensive transthoracic echocardiography within 48 h of admission. Key parameters included LVEF (Simpson's biplane method), Left Atrial Volume Index (LAVI), and PASP estimated from the tricuspid regurgitation velocity plus right atrial pressure. Right ventricular function was assessed using Tricuspid Annular Plane Systolic Excursion (TAPSE) and RV fractional area change. RV-Pulmonary Artery coupling was estimated using the TAPSE/PASP ratio, a validated prognostic marker in patients with heart failure and pulmonary hypertension (21–23).

### Follow-up and outcomes

The primary endpoint was all-cause mortality. The secondary endpoint was rehospitalization for worsening heart failure. Follow-up data were obtained from clinic visits, readmission records, and linkage with the national death registry. The follow-up period extended from the date of index discharge until death or the study censorship date. Follow-up therapies such as catheter ablation, antiarrhythmic drug initiations, or device implantations (ICD/PM) were systematically recorded.

### Statistical analysis

Continuous variables with normal distribution were expressed as mean ± standard deviation (SD) and compared using ANOVA. Non-normally distributed variables were presented as median [interquartile range (IQR)] and compared using the Kruskal–Wallis test. Categorical variables were presented as frequencies (%) and compared using the Chi-square test.

Multinomial logistic regression was performed to identify independent risk factors for each arrhythmia subtype, using the “No Arrhythmia” group as the reference. Variables with a *p*-value < 0.10 in univariate analysis were entered into the multivariate model. Results are reported as Odds Ratios (OR) with 95% Confidence Intervals (CI).

Survival probabilities were estimated using the Kaplan–Meier method and compared using the Log-rank test. To determine the independent prognostic impact of arrhythmia subtypes, multivariate Cox proportional hazards regression models were constructed, adjusting for age, sex, NYHA class, LVEF, PASP, renal function, ischemic cardiomyopathy, and guideline-directed medical therapy (GDMT). The multivariable Cox proportional hazards models were updated to include NT-proBNP levels and device therapy (ICD/PM implantation) during follow-up as time-dependent covariates to minimize residual confounding. To address potential overlap, a secondary analysis treating arrhythmias as non-mutually exclusive time-dependent covariates was also performed. The assumption of proportional hazards was verified using Schoenfeld residuals. A two-sided *p*-value < 0.05 was considered statistically significant. Analysis was performed using R software version 4.5.1 (R Foundation for Statistical Computing, Vienna, Austria).

## Results

### Baseline characteristics of the study cohort

From an initial screening of 2,850 patients, 1,530 patients met the specific criteria for PH-LHF and were included in the analysis (Figure 1). A subset of patients (*n* = 320, 20.9%) underwent right heart catheterization (RHC) to definitively establish the hemodynamic phenotype. The average duration of continuous telemetry monitoring across the cohort was 4.5 days. The mean age was 68.1 ± 11.5 years, 54.9% were male, and the mean LVEF was 38.2 ± 10.8%. The burden of comorbidities was high, with 61.1% hypertensive and 26.5% having CKD. Based on rhythm monitoring, the distribution of arrhythmia phenotypes was: No Arrhythmia (31.1%), Atrial Tachyarrhythmias (42.2%), Ventricular Tachyarrhythmias (18.0%), and Bradyarrhythmias (8.7%). While a hierarchical classification was utilized for primary survival analysis to stratify based on the most prognostically severe rhythm disturbance, we recognized the clinical importance of overlapping arrhythmias. As detailed in Supplementary Table S1, 15% of patients in the VT group also had documented AT.

Detailed baseline characteristics stratified by arrhythmia group are presented in Table 1. Significant heterogeneity was observed across phenotypes. Patients in the **AT group** were significantly older (72.5 ± 9.9 years) and had the highest prevalence of hypertension (72.1%), consistent with a phenotype of stiff left atrial syndrome. Conversely, the **VT group** comprised more males (67.0%) and exhibited the most advanced heart failure profile, characterized by the lowest LVEF (31.5 ± 9.2%), the highest NT-proBNP levels (median 3900 pg/mL), and the most severe hemodynamic impairment (highest PASP: 57.1 ± 14.5 mmHg). The **BA group** had the highest prevalence of CKD (45.1%), suggesting a link between renal dysfunction and conduction system degeneration.

**Table 1:** Baseline demographic and clinical characteristics stratified by arrhythmia subtype (*N* = 1,530).

Variable	Total Cohort (*N* = 1,530)	No Arrhythmia (*N* = 476)	Atrial Tachy. (*N* = 645)	Ventricular Tachy. (*N* = 276)	Bradyarrhythmia (*N* = 133)	*P*-value
Age, years	68.1 ± 11.5	64.2 ± 10.4	72.5 ± 9.9	65.8 ± 12.1	69.8 ± 11.2	<0.001
Male sex, *n* (%)	840 (54.9)	250 (52.5)	325 (50.4)	185 (67.0)	80 (60.2)	<0.001
Hypertension, *n* (%)	935 (61.1)	275 (57.8)	465 (72.1)	140 (50.7)	55 (41.4)	<0.001
Chronic Kidney Disease, *n* (%)	405 (26.5)	95 (20.0)	180 (27.9)	70 (25.4)	60 (45.1)	<0.001
Ischemic Cardiomyopathy, *n* (%)	680 (44.4)	190 (39.9)	260 (40.3)	170 (61.6)	60 (45.1)	<0.001
HF Phenotype, *n* (%)						<0.001
HFrEF (LVEF ≤ 40%)	810 (52.9)	215 (45.2)	310 (48.1)	220 (79.7)	65 (48.9)	
HFmrEF (LVEF 41–49%)	430 (28.1)	155 (32.6)	185 (28.7)	45 (16.3)	45 (33.8)	
HFpEF (LVEF ≥ 50%)	290 (19.0)	106 (22.3)	150 (23.3)	11 (4.0)	23 (17.3)	
LVEF, %	38.2 ± 10.8	41.2 ± 9.9	40.1 ± 10.2	31.5 ± 9.2	37.5 ± 10.5	<0.001
LAVI, ml/m²	42.8 ± 9.1	36.5 ± 7.5	48.5 ± 9.6	39.8 ± 8.4	38.5 ± 8.1	<0.001
PASP, mmHg	48.9 ± 12.6	44.5 ± 8.8	47.5 ± 11.5	57.1 ± 14.5	46.5 ± 10.8	<0.001
TAPSE, mm	17.1 ± 3.9	18.4 ± 3.2	17.0 ± 3.6	14.0 ± 4.2	17.4 ± 4.0	<0.001
NT-proBNP, pg/mL (median)	2,500	1,850	2,350	3,900	2,150	<0.001
Beta-blockers, *n* (%)	1,120 (73.2)	375 (78.8)	460 (71.3)	210 (76.1)	75 (56.4)	<0.001
Antiarrhythmics, *n* (%)	485 (31.7)	45 (9.5)	275 (42.6)	145 (52.5)	20 (15.0)	<0.001
Amiodarone, *n* (%)	350 (22.9)	20 (4.2)	200 (31.0)	125 (45.3)	5 (3.8)	<0.001
SGLT2 inhibitors, *n* (%)	790 (51.6)	255 (53.6)	320 (49.6)	155 (56.2)	60 (45.1)	0.085

Values are mean ± SD, *n* (%), or median.

Significant differences were observed regarding heart failure etiology and pharmacological therapies. Ischemic cardiomyopathy was most prevalent in the VT group (61.6%) compared to the AT (40.3%) and NA (39.9%) groups. Concordantly, the VT cohort was predominantly characterized by HFrEF (79.7%). Regarding antiarrhythmic therapy, amiodarone was the most frequently prescribed agent across the cohort (22.9%), particularly in the VT (45.3%) and AT (31.0%) groups, while beta-blocker utilization was highest in the NA group (78.8%).

### Association between PH severity and arrhythmia Spectrum

**Figure 2:**
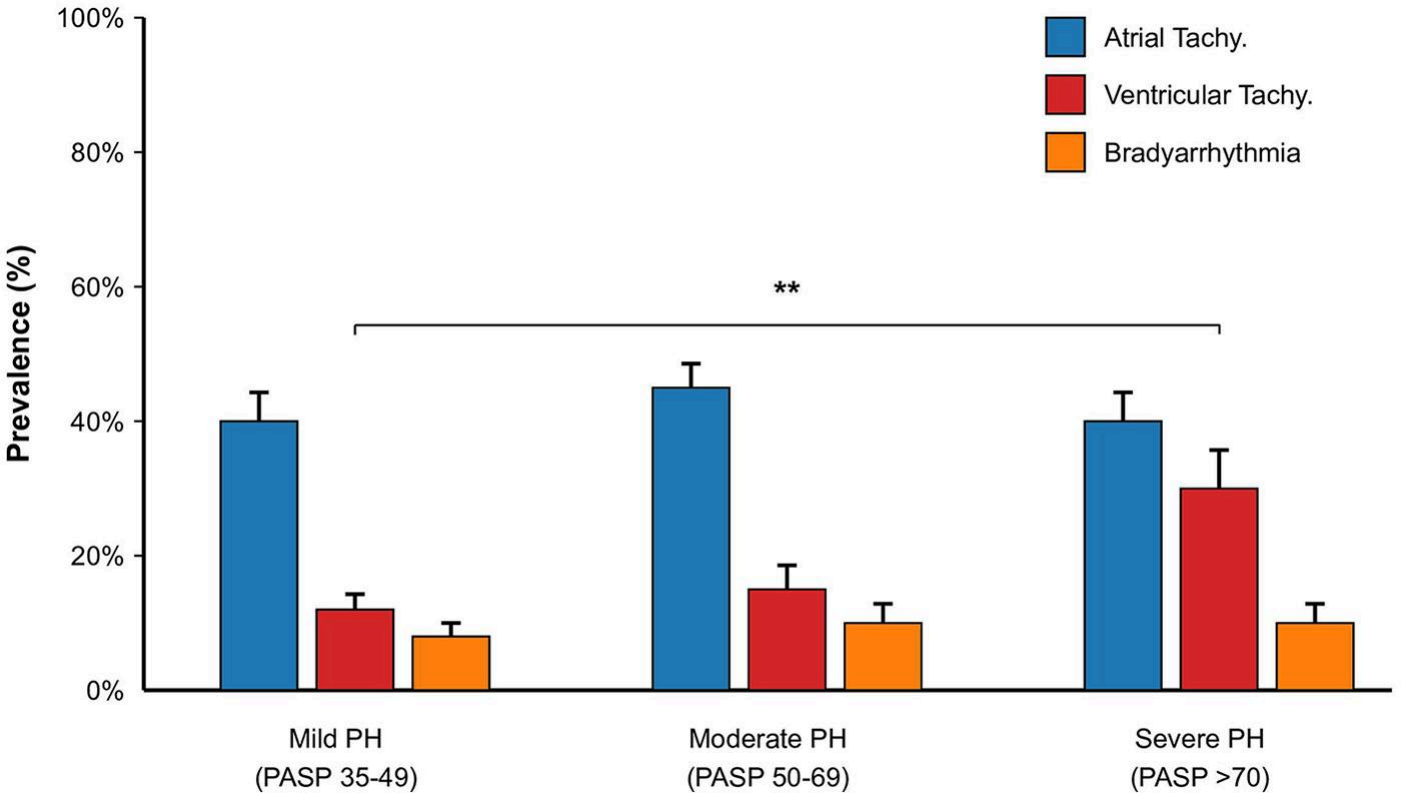
Distribution of arrhythmia types by PH severity. Bar chart showing the prevalence of each arrhythmia subtype across mild (PASP 35–49 mmHg), moderate (PASP 50–69 mmHg), and severe (PASP >70 mmHg) PH groups. Error bars represent standard error. ** Indicates *P* < 0.01 for trend.

We observed a graded relationship between the severity of pulmonary hypertension and the distribution of arrhythmia subtypes. As illustrated in Figure 2, the prevalence of VT rose disproportionately as PASP increased. In patients with mild PH (PASP 35–49 mmHg), VT accounted for only 12% of cases. However, in those with severe PH (PASP >70 mmHg), the prevalence of VT surged to 30%, surpassing the “No Arrhythmia” group. This suggests a dose-response relationship between right ventricular afterload and ventricular electrical instability.

### Phenotype-Specific risk factors

Multivariate logistic regression (Table 2) identified divergent risk factors for each arrhythmia type:

- **Drivers of Atrial Tachyarrhythmias:** The strongest independent predictor was Left Atrial Volume Index (LAVI) >40 ml/m² (OR 2.41, 95% CI 1.85–3.15), confirming that AT in PH-LHF is primarily a consequence of LA stretch. Advanced age (OR 1.05 per year) was also significant. Interestingly, PASP was a moderate predictor, likely serving as a surrogate for chronic LA pressure transmission and elevated left-sided filling pressures (21).
- **Drivers of Ventricular Tachyarrhythmias:** Unlike AT, VT was strongly linked to Right Ventricular parameters. Severe RV dysfunction (TAPSE <16 mm) was the most potent predictor (OR 3.25, 95% CI 2.38–4.45), outperforming LVEF (OR 2.25). Male sex was also an independent risk factor. This pattern strongly supports the central role of RV dysfunction and impaired RV-pulmonary arterial coupling in the arrhythmogenesis of this population, as reflected in prognostic indices like the TAPSE/sPAP ratio (24–26). Furthermore, ischemic cardiomyopathy emerged as a significant independent predictor of VT (OR 2.15, 95% CI 1.50–3.10, *p* < 0.001), reflecting the potent arrhythmogenic substrate of myocardial scar tissue.
- **Drivers of Bradyarrhythmias:** CKD (eGFR <45 ml/min) was the dominant risk factor (OR 1.92), followed by digoxin use and male sex. This profile differs markedly from the tachyarrhythmia groups.

**Table 2:** Multivariate logistic regression analysis for risk factors of specific arrhythmia types.

Variable	Atrial Tachyarrhythmia (vs. No Arrhythmia)		Ventricular Tachyarrhythmia (vs. No Arrhythmia)		Bradyarrhythmia (vs. No Arrhythmia)	
	OR (95% CI)	*P*-value	OR (95% CI)	*P*-value	OR (95% CI)	*P*-value
Age (per 1 year)	1.05 (1.03–1.07)	<0.001	1.01 (0.99–1.03)	0.210	1.03 (1.01–1.06)	0.018
Male Sex	0.90 (0.72–1.12)	0.345	1.65 (1.22–2.25)	0.001	1.52 (1.05–2.20)	0.026
Ischemic Cardiomyopathy	1.15 (0.85–1.55)	0.350	2.15 (1.50–3.10)	<0.001	1.25 (0.80–1.95)	0.320
LAVI >40 ml/m²	2.41 (1.85–3.15)	<0.001	1.15 (0.82–1.62)	0.420	1.08 (0.70–1.65)	0.735
LVEF <35%	1.12 (0.85–1.48)	0.410	2.25 (1.65–3.08)	<0.001	1.25 (0.82–1.92)	0.305
TAPSE <16 mm	1.18 (0.90–1.55)	0.225	3.25 (2.38–4.45)	<0.001	1.12 (0.72–1.75)	0.615
eGFR <45 ml/min	1.35 (1.02–1.78)	0.035	1.42 (1.01–2.02)	0.045	1.92 (1.35–2.75)	<0.001

Adjusted for relevant baseline characteristics.

### Prognostic impact: survival and rehospitalization

**Figure 3:**
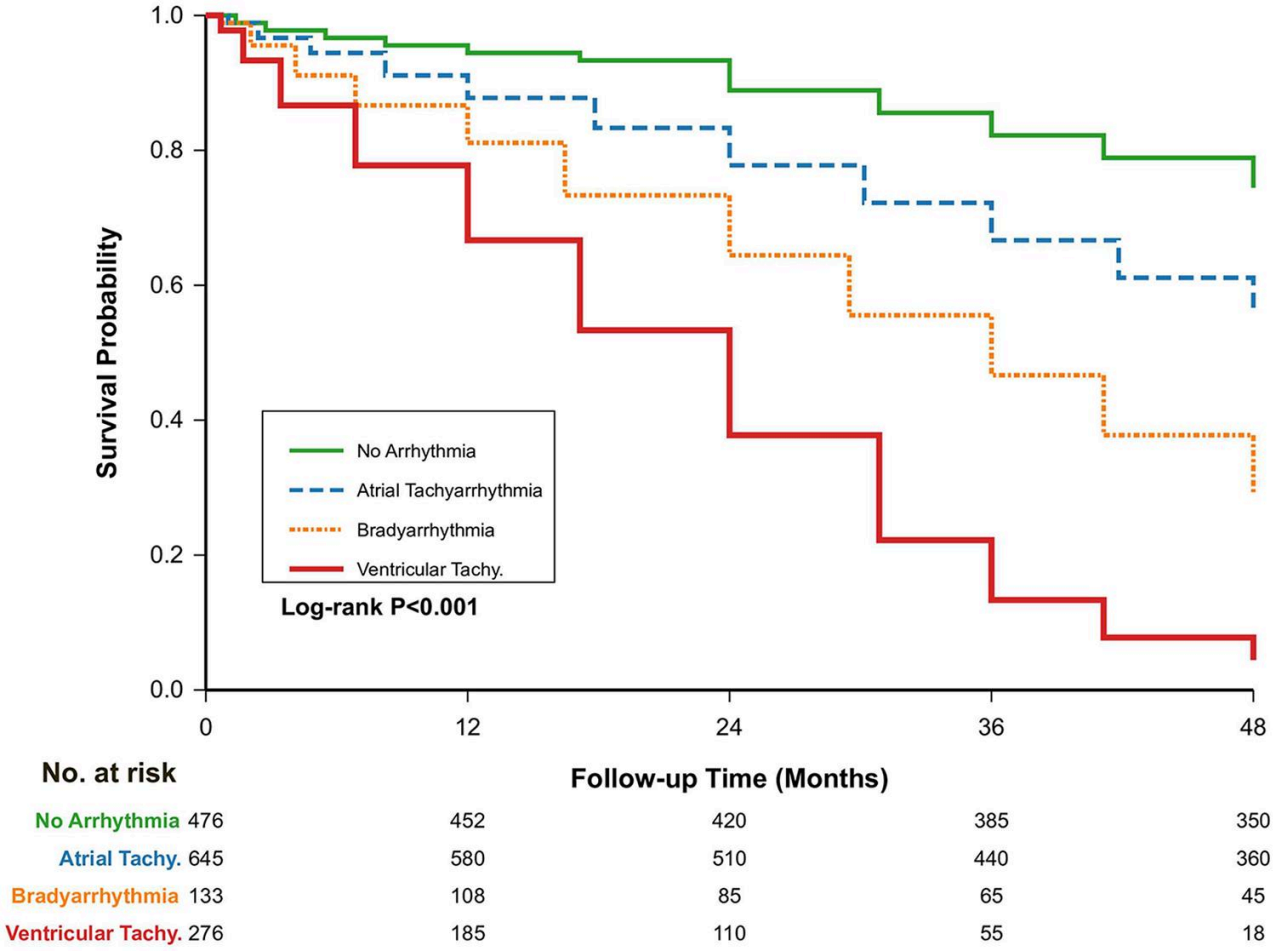
Kaplan–meier survival analysis. Time-to-event curves for all-cause mortality stratified by arrhythmia subtype over 48 months. The Ventricular Tachyarrhythmia (VT) group demonstrates significantly worse survival compared to all other groups (Log-rank *p* < 0.001). Numbers at risk are displayed below the *x*-axis.

Over a median follow-up of 38 months, 435 deaths (28.4%) and 725 HF rehospitalizations (47.4%) were recorded. Kaplan–Meier survival curves (Figure 3) revealed highly significant differences in outcomes (Log-rank *p* < 0.001). The VT group exhibited the steepest decline in survival, with a 3-year survival rate of approximately 55%, compared to nearly 80% in the NA group. The BA group also showed poor survival, intermediate between VT and AT.

**Figure 4:**
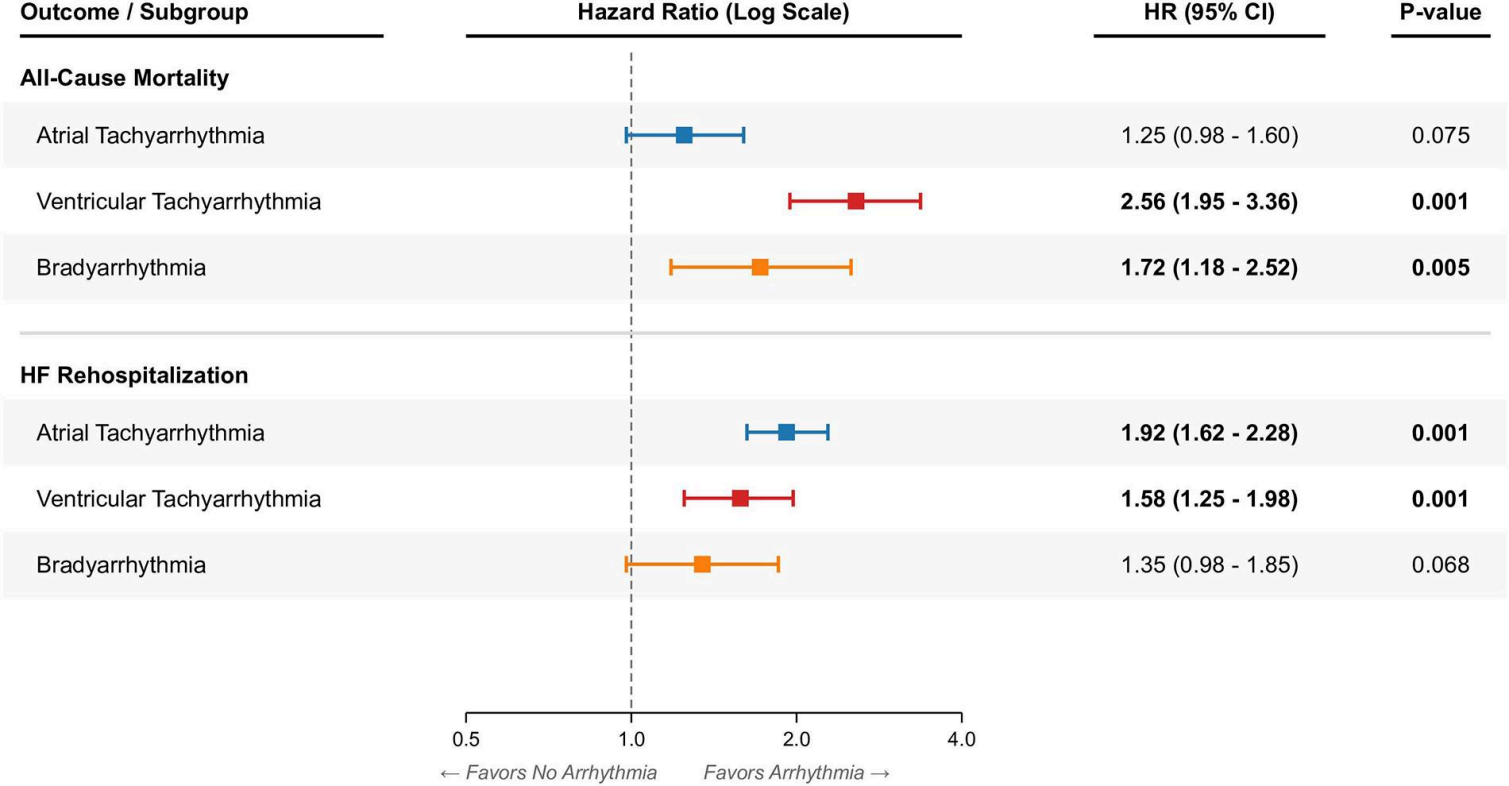
Forest plot of hazard ratios. Visualization of the Multivariate Cox Proportional Hazards model results for All-Cause Mortality and HF Rehospitalization. Points represent Hazard Ratios and horizontal lines represent 95% Confidence Intervals.

Multivariate Cox proportional hazards analysis (Figure 4 and Table 3) confirmed these trends after adjusting for confounders, including updated adjustments for NT-proBNP, ischemic cardiomyopathy, and device therapies. The VT phenotype remained the strongest independent predictor of all-cause mortality (HR 2.56, 95% CI 1.95–3.36, *p* < 0.001). Secondary analysis treating arrhythmias as non-mutually exclusive time-dependent covariates corroborated the independent prognostic weight of VT on mortality. Ischemic cardiomyopathy also remained an independent predictor of all-cause mortality (HR 1.45, 95% CI 1.20–1.75, *p* < 0.001). While the AT phenotype had a modest impact on mortality (HR 1.25, *p* = 0.075), it was the most powerful driver of heart failure rehospitalization (HR 1.92, 95% CI 1.62–2.28, *p* < 0.001), reflecting the symptomatic burden of loss of AV synchrony and rapid ventricular rates.

**Table 3:** Multivariate Cox proportional hazards analysis for clinical outcomes.

Variable	All-Cause Mortality		HF Rehospitalization	
	HR (95% CI)	*P*-value	HR (95% CI)	*P*-value
Arrhythmia Type (Ref: None)				
Atrial Tachyarrhythmia	1.25 (0.98–1.60)	0.075	1.92 (1.62–2.28)	<0.001
Ventricular Tachyarrhythmia	2.56 (1.95–3.36)	<0.001	1.58 (1.25–1.98)	<0.001
Bradyarrhythmia	1.72 (1.18–2.52)	0.005	1.35 (0.98–1.85)	0.068
Other Key Predictors				
Ischemic Cardiomyopathy	1.45 (1.20–1.75)	<0.001	1.30 (1.10–1.55)	0.002

Adjusted for age, sex, NYHA class, LVEF, PASP, renal function, GDMT, NT-proBNP levels, ischemic etiology, and device therapy (ICD/PM) during follow-up as time-dependent covariates.

## Discussion

To our knowledge, this is one of the largest studies to date specifically examining the interplay between arrhythmia subtypes and the PH-LHF phenotype. By moving beyond the binary “arrhythmia yes/no” classification, our study provides three novel insights that have direct clinical implications: (1) the arrhythmic burden in PH-LHF is exceptionally high (∼70%) and diverse; (2) risk factors are anatomically distinct, with RV dysfunction driving malignant ventricular arrhythmias; and (3) prognosis is phenotype-dependent, with VT dictating survival and AT dictating healthcare utilization.

### The “failing right heart” as a driver of ventricular arrhythmias

Traditionally, VT risk stratification in HF has focused on the left ventricle (LVEF, scar burden). However, our findings powerfully highlight the role of the right ventricle in PH-LHF. The strong association between TAPSE <16 mm and VT (OR 3.25) aligns with emerging mechanistic data. As pulmonary arterial pressure and right ventricular afterload rise, the RV undergoes hypertrophy and dilation. This results in mechanical wall stress, subendocardial ischemia (due to increased wall tension and reduced perfusion pressure), and interstitial fibrosis (27). Furthermore, “septal bowing” due to pressure overload can disrupt the electromechanical continuity of the ventricles (28, 29). Our observational findings are hypothesis-generating and suggest a pathophysiological link that warrants confirmation in prospective, imaging-based studies. Our data suggests that in PH-LHF, the RV is not an innocent bystander but an active source of arrhythmogenicity. This challenges current ICD guidelines, which do not strictly incorporate RV function.

It is important to highlight that the VT population represented a more advanced heart failure cohort, with a striking predominance of HFrEF and ischemic cardiomyopathy. As confirmed by our multivariable analysis, ischemic etiology is a powerful, independent predictor of VT, consistent with the known arrhythmogenic risk of myocardial scar (30). However, even after rigorous adjustment for ischemic etiology, LVEF, and NT-proBNP, severe RV dysfunction (TAPSE <16 mm) retained its robust independent association with VT. We propose that RV parameters could serve as tools for risk enrichment in this population, rather than advocating for immediate modifications to ICD guidelines. Prospective validation is essential before clinical adoption. Therefore, these findings should be viewed as hypothesis-generating, prompting future trials to evaluate whether RV-focused criteria can optimize ICD candidate selection (11, 31, 32).

### Atrial fibrillation: A barometer of left heart compliance

We found AT to be the most prevalent arrhythmia (42.2%). Our data confirms that LA volume is the primary determinant, but uniquely, we found that AT was the strongest driver of rehospitalization (HR 1.92). In PH-LHF, the left ventricle is often stiff (diastolic dysfunction). These patients are critically dependent on the “atrial kick” for ventricular filling. The onset of AF leads to a sudden loss of this contribution, causing an abrupt rise in LA pressure that is immediately transmitted to the pulmonary bed, precipitating “flash” pulmonary edema (33). This mechanism explains why AF drives hospitalization so potently in this specific cohort compared to general heart failure populations. This supports the strategy of early rhythm control (ablation) in PH-LHF patients, not just for symptom relief, but to prevent recurrent hemodynamic decompensation, a concept supported by the recent CASTLE-HTx trial results, which demonstrated a significant reduction in a composite endpoint of death, left ventricular assist device implantation, or urgent heart transplantation with catheter ablation (34).

### Bradyarrhythmias and the “uremic-septal” axis

The 8.7% prevalence of significant bradyarrhythmias is noteworthy. The strong link to CKD suggests a systemic fibrotic process affecting both the kidneys and the cardiac conduction system. Additionally, the mechanical strain of PH on the interventricular septum—where the bundle branches reside—may accelerate conduction disease. The high mortality in this group (HR 1.72) is concerning. It implies that “chronotropic incompetence” limits the cardiac output reserve necessary to overcome high pulmonary vascular resistance during daily activity, leading to progressive low-output failure. Recent evidence in HFpEF patients indicates that chronotropic incompetence is independently associated with elevated pulmonary capillary wedge pressure, impaired right ventricle–arterial coupling, reduced exercise capacity, and a significantly increased risk of cardiovascular death or hospitalization (HR 1.725) (35). This supports the pathophysiological link where inadequate heart rate response compromises the cardiac output needed to meet the demands imposed by pulmonary hypertension, thereby driving adverse outcomes.

### Clinical implications

Our findings argue for a tailored management approach in PH-LHF:

1. **Screening:** Patients with PH-LHF and echocardiographic signs of RV dysfunction (TAPSE <16 mm) should undergo prolonged rhythm monitoring (e.g., ILR) to detect occult VT.
2. **Prevention:** Aggressive management of PVR and congestion (diuretics, potentially SGLT2 inhibitors) may be anti-arrhythmic by unloading the RV.
3. **Therapy:** For AT, rhythm control should be prioritized to prevent hospitalization. For VT, the threshold for ICD might need to be re-evaluated to include RV parameters.

### Limitations

This study is retrospective, relying on clinically indicated monitoring which may underestimate paroxysmal arrhythmias. The average duration of continuous telemetry was 4.5 days. We acknowledge that this may underestimate the true burden of paroxysmal arrhythmias. We did not have uniform genetic testing or cardiac MRI data to characterize myocardial fibrosis tissue. Additionally, while we used strict echocardiographic definitions for PH, right heart catheterization was not performed in all patients, limiting our ability to strictly differentiate between isolated post-capillary PH (Ipc-PH) and Cpc-PH. While only 320 patients (20.9%) underwent right heart catheterization (RHC) to definitively diagnose combined pre- and post-capillary PH (Cpc-PH), the echocardiographic parameters utilized serve as surrogates for right ventricular strain. Furthermore, it is important to acknowledge that the strong association between VT and mortality may partly reflect advanced heart failure severity or unmeasured myocardial scar burden that could not be fully adjusted for in our models. Despite robust multivariable adjustments, residual confounding by overall heart failure severity cannot be entirely ruled out. Finally, while we recorded the baseline use of antiarrhythmic agents, specifically noting the frequent use of amiodarone in the VT and AT groups, detailed data regarding cumulative dosing, duration of therapy, and subsequent modifications or discontinuations were not fully captured. Different antiarrhythmic strategies may significantly influence long-term outcomes, and this remains a potential unmeasured confounder.

## Conclusion

In patients with PH-LHF, arrhythmias are a defining feature of the disease trajectory. The type of arrhythmia is dictated by the underlying structural phenotype: LA remodeling drives atrial arrhythmias which cause morbidity, while RV remodeling drives ventricular arrhythmias which cause mortality. Recognizing this divergence is the first step toward precision medicine in this complex syndrome. Future trials should investigate whether RV-guided risk stratification can improve survival in this high-risk population.

## Data Availability

The original contributions presented in the study are included in the article/Supplementary Material, further inquiries can be directed to the corresponding author/s.

## References

[1] Humbert M Kovacs G Hoeper MM Badagliacca R Berger RMF Brida M 2022 ESC/ERS guidelines for the diagnosis and treatment of pulmonary hypertension. Eur Respir J. (2023) 61(1):2200879. 10.1183/13993003.00879-2022 36028254

[2] Simonneau G Montani D Celermajer DS Denton CP Gatzoulis MA Krowka M Haemodynamic definitions and updated clinical classification of pulmonary hypertension. Eur Respir J. (2019) 53(1):1801913. 10.1183/13993003.01913-2018 30545968 PMC6351336

[3] Riccardi M Pagnesi M Sciatti E Lombardi CM Inciardi RM Metra M Combined pre- and post-capillary pulmonary hypertension in left heart disease. Heart Fail Rev. (2023) 28(1):137–48. 10.1007/s10741-022-10251-9 35650331

[4] Rosenkranz S Gibbs JS Wachter R De Marco T Vonk-Noordegraaf A Vachiéry JL. Left ventricular heart failure and pulmonary hypertension. Eur Heart J. (2016) 37(12):942–54. 10.1093/eurheartj/ehv512 26508169 PMC4800173

[5] Duran A Mandras S. Pulmonary hypertension in heart failure. Curr Opin Cardiol. (2021) 36(2):205–10. 10.1097/HCO.0000000000000834 33394713

[6] Fingrova Z Ambroz D Jansa P Kuchar J Lindner J Kunstyr J The prevalence and clinical outcome of supraventricular tachycardia in different etiologies of pulmonary hypertension. PLoS One. (2021) 16(1):e0245752. 10.1371/journal.pone.0245752 33471824 PMC7817034

[7] Sartipy U Dahlström U Fu M Lund LH. Atrial fibrillation in heart failure with preserved, mid-range, and reduced ejection fraction. JACC Heart Fail. (2017) 5(8):565–74. 10.1016/j.jchf.2017.05.001 28711451

[8] Anand S Cronin EM. Arrhythmias in patients with pulmonary hypertension and right ventricular failure: importance of rhythm control strategies. J Clin Med. (2024) 13(7):1866. 10.3390/jcm13071866 38610631 PMC11012772

[9] Joglar JA Chung MK Armbruster AL Benjamin EJ Chyou JY Cronin EM 2023 ACC/AHA/ACCP/HRS guideline for the diagnosis and management of atrial fibrillation: a report of the American College of Cardiology/American Heart Association joint committee on clinical practice guidelines. J Am Coll Cardiol. (2024) 83(1):109–279. 10.1016/j.jacc.2023.08.017 38043043 PMC11104284

[10] Abouzaid A Ali K Jatoi S Ahmed M Ahmad G Nazim A Cardiac arrhythmias in pulmonary arterial hypertension and chronic thromboembolic pulmonary hypertension: mechanistic insights, pathophysiology, and outcomes. Ann Noninvasive Electrocardiol. (2024) 29(5):e70010. 10.1111/anec.13115 39205610 PMC11358588

[11] Zeppenfeld K Tfelt-Hansen J de Riva M Winkel BG Behr ER Blom NA 2022 ESC guidelines for the management of patients with ventricular arrhythmias and the prevention of sudden cardiac death. Eur Heart J. (2022) 43(40):3997–4126. 10.1093/eurheartj/ehac262 36017572

[12] Motoishi H Uesawa Y Ishii-Nozawa R. Evaluation of *β*-blocker-induced bradyarrhythmia using an analysis of the Japanese adverse drug event report database. Biol Pharm Bull. (2024) 47(10):1668–74. 10.1248/bpb.b24-00305 39443084

[13] von Elm E Altman DG Egger M. The strengthening the reporting of observational studies in epidemiology (STROBE) statement: guidelines for reporting observational studies. J Clin Epidemiol. (2007) 60(6):344–9. 10.1016/j.jclinepi.2007.01.028 18313558

[14] Galiè N Humbert M Vachiery JL Gibbs S Lang I Torbicki A 2015 ESC/ERS guidelines for the diagnosis and treatment of pulmonary hypertension. Rev Esp Cardiol. (2016) 69(2):177. 10.1016/j.rec.2016.01.002 26837729

[15] Omote K Sorimachi H Obokata M Reddy YNV Verbrugge FH Omar M Pulmonary vascular disease in pulmonary hypertension due to left heart disease: pathophysiologic implications. Eur Heart J. (2022) 43(36):3417–31. 10.1093/eurheartj/ehac184 35796488 PMC9794188

[16] Yamabe S Dohi Y Fujisaki S Higashi A Kinoshita H Sada Y Prognostic factors for survival in pulmonary hypertension due to left heart disease. Circ J. (2016) 80(1):243–9. 10.1253/circj.CJ-15-0708 26581623

[17] Al-Khatib SM Stevenson WG Ackerman MJ Bryant WJ Callans DJ Curtis AB 2017 AHA/ACC/HRS guideline for management of patients with ventricular arrhythmias and the prevention of sudden cardiac death: a report of the American College of Cardiology/American Heart Association task force on clinical practice guidelines and the heart rhythm society. J Am Coll Cardiol. (2018) 72(14):e91–e220. 10.1016/j.jacc.2017.10.054 29097296

[18] Kirchhof P Benussi S Kotecha D Ahlsson A Atar D Casadei B 2016 ESC guidelines for the management of atrial fibrillation developed in collaboration with EACTS. Eur Heart J. (2016) 37(38):2893–962. 10.1093/eurheartj/ehw210 27567408

[19] Kotecha D Bunting KV Gill SK Mehta S Stanbury M Jones JC Effect of digoxin vs bisoprolol for heart rate control in atrial fibrillation on patient-reported quality of life: the RATE-AF randomized clinical trial. Jama. (2020) 324(24):2497–508. 10.1001/jama.2020.23138 33351042 PMC7756234

[20] Kusumoto FM Schoenfeld MH Barrett C Edgerton JR Ellenbogen KA Gold MR 2018 ACC/AHA/HRS guideline on the evaluation and management of patients with bradycardia and cardiac conduction delay: a report of the American College of Cardiology/American Heart Association task force on clinical practice guidelines and the heart rhythm society. Circulation. (2019) 140(8):e382–482. 10.1161/CIR.0000000000000628 30586772

[21] Guazzi M Bandera F Pelissero G Castelvecchio S Menicanti L Ghio S Tricuspid annular plane systolic excursion and pulmonary arterial systolic pressure relationship in heart failure: an index of right ventricular contractile function and prognosis. Am J Physiol Heart Circ Physiol. (2013) 305(9):H1373–1381. 10.1152/ajpheart.00157.2013 23997100

[22] Schmeisser A Rauwolf T Groscheck T Kropf S Luani B Tanev I Pressure-volume loop validation of TAPSE/PASP for right ventricular arterial coupling in heart failure with pulmonary hypertension. Eur Heart J Cardiovasc Imaging. (2021) 22(2):168–76. 10.1093/ehjci/jeaa285 33167032

[23] McDonagh TA Metra M Adamo M Gardner RS Baumbach A Böhm M 2021 ESC guidelines for the diagnosis and treatment of acute and chronic heart failure. Eur Heart J. (2021) 42(36):3599–726. 10.1093/eurheartj/ehab368 34447992

[24] de Pinto M Coppi F Spinella A Pagnoni G Morgante V Macripò P The predictive role of the TAPSE/sPAP ratio for cardiovascular events and mortality in systemic sclerosis with pulmonary hypertension. Front Cardiovasc Med. (2024) 11:1430903. 10.3389/fcvm.2024.1430903 39469124 PMC11513352

[25] Lang RM Badano LP Mor-Avi V Afilalo J Armstrong A Ernande L Recommendations for cardiac chamber quantification by echocardiography in adults: an update from the American society of echocardiography and the European association of cardiovascular imaging. J Am Soc Echocardiogr. (2015) 28(1):1–39.e14. 10.1016/j.echo.2014.10.003 25559473

[26] Çolak A Kumral Z Kış M Şentürk B Sezgin D Ömeroğlu Şimşek G The usefulness of the TAPSE/sPAP ratio for predicting survival in medically treated chronic thromboembolic pulmonary hypertension. Turk Kardiyol Dern Ars. (2023) 51(7):470–7. 10.5543/tkda.2023.78074 37861261

[27] Tanaka Y Takase B Yao T Ishihara M. Right ventricular electrical remodeling and arrhythmogenic substrate in rat pulmonary hypertension. Am J Respir Cell Mol Biol. (2013) 49(3):426–36. 10.1165/rcmb.2012-0089OC 23600532

[28] Kresoja KP Rosch S Schöber AR Fengler K Schlotter F Bombace S Implications of tricuspid regurgitation and right ventricular volume overload in patients with heart failure with preserved ejection fraction. Eur J Heart Fail. (2024) 26(4):1025–35. 10.1002/ejhf.3195 38462987

[29] Ichimura K Santana EJ Kuznetsova T Cauwenberghs N Sabovčik F Chun L Novel left ventricular mechanical index in pulmonary arterial hypertension. Pulm Circ. (2023) 13(2):e12216. 10.1002/pul2.12216 37063750 PMC10103585

[30] Heidenreich PA Bozkurt B Aguilar D Allen LA Byun JJ Colvin MM 2022 AHA/ACC/HFSA guideline for the management of heart failure: a report of the American College of Cardiology/American Heart Association joint committee on clinical practice guidelines. Circulation. (2022) 145(18):e895–e1032. 10.1161/CIR.0000000000001063 35363499

[31] Cheng WH Chung FP Lin YJ Lo LW Chang SL Hu YF Arrhythmogenic right ventricular cardiomyopathy: diverse substrate characteristics and ablation outcome. Rev Cardiovasc Med. (2021) 22(4):1295–309. 10.31083/j.rcm2204136 34957771

[32] Corrado D van Tintelen PJ McKenna WJ Hauer RNW Anastastakis A Asimaki A Arrhythmogenic right ventricular cardiomyopathy: evaluation of the current diagnostic criteria and differential diagnosis. Eur Heart J. (2020) 41(14):1414–29. 10.1093/eurheartj/ehz669 31637441 PMC7138528

[33] Velliou M Sanidas E Diakantonis A Ventoulis I Parissis J Polyzogopoulou E. The optimal management of patients with atrial fibrillation and acute heart failure in the emergency department. Medicina. (2023) 59(12):2113. 10.3390/medicina59122113 38138216 PMC10744575

[34] Sohns C Fox H Marrouche NF Crijns H Costard-Jaeckle A Bergau L Catheter ablation in End-stage heart failure with atrial fibrillation. N Engl J Med. (2023) 389(15):1380–9. 10.1056/NEJMoa2306037 37634135

[35] Lin TT Chen TY Cheng JF Lin LY Wu CK. Chronotropic incompetence and cardiovascular outcomes in patients with heart failure with preserved ejection fraction. J Am Heart Assoc. (2025) 14(13):e037290. 10.1161/JAHA.124.037290 40530480 PMC12449948

