# Supplementary material for: Risk factors for different types of arrhythmias and their prognostic impact in patients with heart failure and concomitant pulmonary hypertension (PH-LHF): a large-scale retrospective cohort study
Source: Front Cardiovasc Med. 2026 Apr 15;13:1773989. doi: 10.3389/fcvm.2026.1773989 (PMC13124940; doi:10.3389/fcvm.2026.1773989)
Supplement: Supplementary file 1 [file Supplementaryfile1.docx]

**Supplementary Table 1. Overlap of Documented Arrhythmia Subtypes in the Total Cohort (N=1530)**

| **Primary Hierarchical Assignment** | **Total in Group (N)** | **Co-occurring Atrial Tachy. n (%)** | **Co-occurring Ventricular Tachy. n (%)** | **Co-occurring Bradyarrhythmia n (%)** |
| --- | --- | --- | --- | --- |
| No Arrhythmia (NA) | 476 | - | - | - |
| Atrial Tachy. (AT) | 645 | - | N/A* | 52 (8.1%) |
| Ventricular Tachy. (VT) | 276 | 41 (14.9%) | - | 18 (6.5%) |
| Bradyarrhythmia (BA) | 133 | N/A* | N/A* | - |

*N/A indicates not applicable due to hierarchical assignment (e.g., if a BA patient developed AT, they would be reclassified into the AT group for the primary analysis model).
